# Moving forward: Scaling-up the integration of an HIV and hypertension program in Akwa Ibom State, Nigeria

**DOI:** 10.21203/rs.3.rs-3979683/v1

**Published:** 2024-02-28

**Authors:** Shivani Mishra, Angela Aifah, Daniel Henry, Nina Uzoigwe, Emem Udoh, Esther Idang, Jahnavi Munagala, Deborah Onakomaiya, Nafesa Kanneh, Anyiekere Ekanem, Eno Angela Attah, Gbenga Ogedegbe, Dike Ojji

**Affiliations:** New York University Grossman School of Medicine; New York University Grossman School of Medicine; University of Abuja Teaching Hospital; New York University Grossman School of Medicine; University of Abuja Teaching Hospital; University of Abuja Teaching Hospital; New York University Grossman School of Medicine; New York University Grossman School of Medicine; New York University Grossman School of Medicine; University of Uyo Teaching Hospital; Akwa Ibom State Primary Healthcare Development Agency; New York University Grossman School of Medicine; University of Abuja Teaching Hospital

**Keywords:** HIV, Policy, Non-communicable diseases, Primary healthcare, Capacity building

## Abstract

**Background:**

As people living with HIV experience increased life expectancy, there is a growing concern about the burden of comorbid non-communicable diseases, particularly hypertension. This policy brief describes the current policy landscape in Akwa Ibom State, Nigeria, the research activities, and five policy recommendations rooted in an ongoing research study designed to integrate hypertension management into HIV care across primary health centers in the state.

**Analysis:**

The policy brief was developed in four steps: review of existing policies, using the reviewed policies to inform research activities, solicitation of stakeholder recommendations via focus group discussions, and formulation of the resulting five policy recommendations for integrating hypertension management into HIV care programs in Akwa Ibom. The key analysis for this brief emerged from the thematic analyses of stakeholder responses.

**Policy Implications:**

The five policy recommendations for integrating hypertension management in HIV care in Akwa Ibom State, Nigeria are: 1) build capacity by leveraging retired community nurses as mentors; 2) emphasize community engagement; 3) develop consistent training programs on hypertension management for health workers; 4) expand health insurance accessibility; and 5) formally integrate hypertension management into primary healthcare centers in Akwa Ibom State.

## BACKGROUND

Over the last 20 years, Nigeria has had one of the highest prevalence of HIV infection in West Africa, and is estimated to have an increase in deaths associated with hypertension as a result of side effects of anti-retroviral treatment.^[1]^ On the other hand, HIV treatment programs have been associated with increased life expectancy among people living with HIV (PLWH).^[2]^ This may explain the higher levels of comorbid non-communicable diseases (NCDs) like hypertension, making its prevention and management a priority. ^[2]^ There is an urgent need to leverage available HIV care systems and resources to address the rising burden of NCDs in PLWH. Our research study, *Managing Hypertension Among People Living with HIV: an Integrated Model (MAP-IT)*, is designed to address this important problem, and its goal is to evaluate the adoption and sustainability of an integrated HIV and hypertension program among PLWH who receive care in primary care centers in Akwa Ibom State^[3]^ - a state with one of the highest burden in Nigeria. Specifically, MAP-IT provides hypertension care to PLWH through the existing HIV care services in primary healthcare centers across Akwa Ibom State.^[3]^ Trained community nurses implement the components of the program, which include identifying, counseling, treating, and referring PLWH with uncontrolled hypertension. The community nurses are then mentored by trained and retired community who serve as practice facilitators. ^[3]^

**Figure 1:**
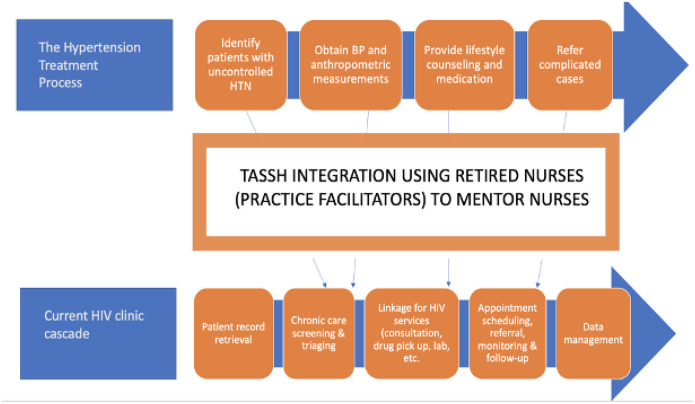
Key components of the MAP-IT study

As part of MAP-IT, we identified key stakeholders within the health system, solicited their feedback, and outlined policy recommendations on how to scale-up current resources needed to integrate hypertension management into HIV care. The key stakeholders engaged at multiple levels include *policymakers, patient advocacy groups*, and *healthcare workers*. Figure 1 summarizes the components of the MAP-IT study.

## ANALYSIS

**Figure 2:**
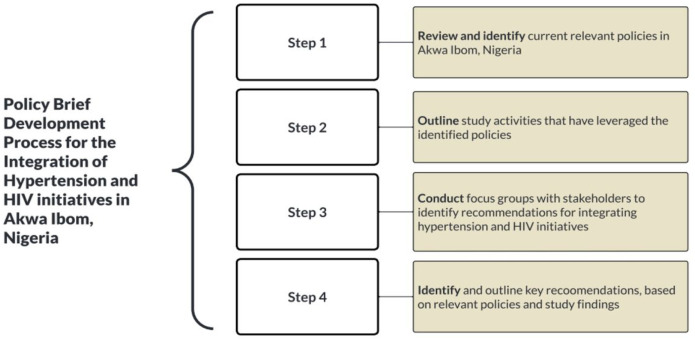
Steps for the development of the policy brief advocating for the integration of hypertension management into HIV care in Akwa Ibom, Nigeria

The development of this policy brief followed four steps. First, we reviewed and identified the current relevant policies. Second, we outlined activities of the MAP-IT’s study that leverage the policies identified. Next, we conducted focus groups with the key stakeholders to identify their recommendations. In the final step, we outlined five policy recommendations for integrating hypertension management into HIV care across primary health centers (PHCs) in Akwa Ibom State. The four steps are summarized in Fig. 2, and details of each step are described further.

### Step 1: Identification of current relevant policies:

The MAP-IT study is focused on two strategies that require task-shifting of hypertension treatment duties to a nurse and implementation of the program in PHCs which otherwise does not provide specialized care for people with NCDs. As such, the three relevant policies that are pertinent to the MAP-IT study are task-shifting policy, the national Hypertension Treatment Protocol, and the State Health Insurance Policy, all described next.

#### 1. The Nigerian Federal Ministry of Health has a task-shifting and sharing policy

Nigeria has an established task-shifting and sharing policy (the ‘Task Shifting and Sharing Policy for Essential Health Care Services in Nigeria) that provides access to vital health services through the efficient use of non-physician health workers to manage high mortality diseases like HIV.^[4]^ By promoting skilled, non-physician workers to perform key tasks including case identification, referrals, initiation of treatment, and routine physical examinations, the policy makes hypertension management integration into HIV care platforms attainable and sustainable. ^[3]^

#### 2. Nigeria has a national Hypertension Treatment Protocol

The national Hypertension Treatment Protocol is a 4-step simplified process for identifying and initiation patients on antihypertension medication treatment, and was developed for use within the primary care system in Nigeria ^[5]^ The protocol, when implemented by community nurses on PLWH in primary care clinics, allows the identification and management of hypertension among the population.

#### 3. The Akwa Ibom State has established a State Health Insurance Agency

In February 2023, the Akwa Ibom State Health Insurance Agency was launched, which aims to achieve universal health coverage in the state by providing financial access to qualitative, affordable, equitable and sustainable health care services.^[6]^ Such ongoing efforts to achieve universal health coverage is important to advance the integration of hypertension treatment into HIV care because it facilitates collaboration between healthcare providers in the state as well as cost reduction associated by managing both conditions.^[7]^

### Step 2: MAP-IT study activities that leveraged identified policies

#### 1. Conducting assessments

To better understand the needs of our partners and align with Akwa Ibom State Health Insurance Agency’s priority of achieving sustainable health services in the state, MAP-IT assessed the facilities and their capacity for change by conducting in-depth interviews and holding a stakeholder engagement meeting as well as five community advisory board meetings.

#### 2. Connecting communities to clinics providing HIV and hypertension integrated care

Following the assessments and after identifying gaps in services, **43** onsite training sessions for community nurses and other healthcare workers have been conducted. The study currently provides hypertension care, including free antihypertensive medications, for over **1746** persons living with HIV.

#### 3. Providing supplies

**254** Semi-automated sphygmomanometers were distributed to primary health care centers and HIV support groups with constant provision and supply of batteries for their operation. Anthropometric devices, including weighing scales, stadiometers, and tapes, were also provided to these facilities where they were not available.

#### 4. Building capacity

Using models developed from the WHO HEARTS Package and Nigerian Hypertension Treatment Protocol^[5]^, we carry out structured interval trainings on simplified management of hypertension for all community nurses, community pharmacy technicians, case managers and practice facilitators who are working in the **30** primary healthcare where the MAP-IT study is being conducted. In addition, we are building capacity among physician and non-physician healthcare workers working in Akwa Ibom State in the management of hypertension. As of January 2024, we have trained over **790** of such personnel comprising community nurses working in all primary care facilities in Akwa Ibom State, nursing tutors in school of nursing and midwifery, 135 community pharmacists and over **205** primary care physicians.

#### 5. Providing incentives

Honorariums are provided to community nurses and pharmacists, while study participants receive transport stipends.

Notably, an important aspect of task-shifting policy is training of community, provision of supplies to advance hypertension care, provision of equipment for blood pressure assessment as well as other equipment like weighing scales and tape rule for cardiovascular risk identification. As such, a comprehensive assessment of the practice capacity of each PHC is crucial to help identify the gaps and what is needed.

### Step 3: Conduct focus groups with stakeholders to identify recommendations for integrating hypertension and HIV initiatives

In November 2022, three groups of stakeholders (8 healthcare providers, 7 patients and beneficiaries and 6 policymakers) participated in three separate 60–90-minute focus group discussions led by trained research staff. Semi-structured questions centered on recommendations for scaling-up the integration of hypertension management in HIV care were asked. The focus groups were audiotaped and transcribed. An inductive thematic analysis of the transcribed discussions was conducted, with results triangulated among three trained research staff to identify stakeholder-informed recommendations for scaling-up and disseminating the integration of hypertension management in HIV care across PHCs.

## POLICY IMPLICATIONS

**Figure 3:**
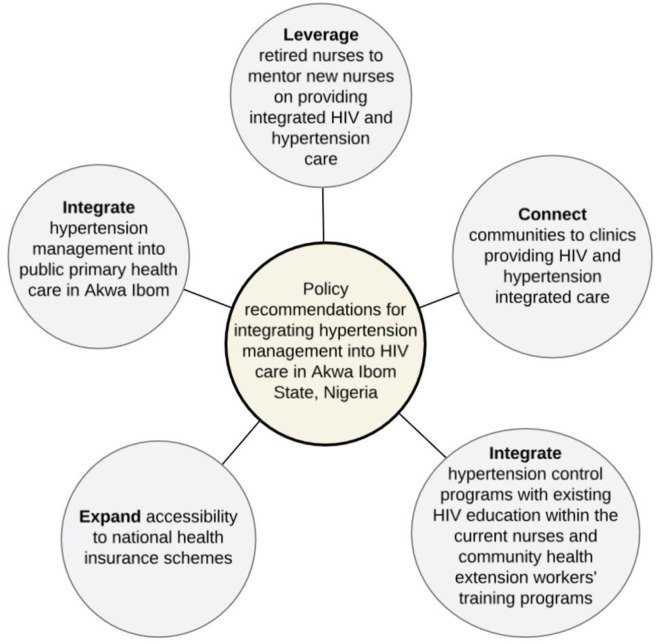
Policy recommendations for integrating hypertension management into HIV care in Akwa Ibom, Nigeria

Figure 3 summarizes the five policy recommendations that emerged from the focus group discussions and the inductive analyses of the transcripts.

### Recommendation 1: Leverage retired nurses to mentor community nurses on providing integrated HIV and hypertension care

Identifying retired community as valuable sources of knowledge maximizes the potential of resources within the country’s health system and provides a dignified pathway to contribution to retirees. Our preliminary findings show that training and engaging retired community nurses will enhance the management of hypertension among PLWH.

### Recommendation 2: Connect communities to clinics providing HIV and hypertension integrated care

To accomplish this, it will be necessary to include **all** community-based organizations and groups to sensitize communities on the burden of hypertension among PLWH and link communities to closest clinics. These community-based organizations include but are certainly not limited to: Network of People Living with HIV/Aids in Nigeria, and Association of Women Living with HIV/AIDS in Nigeria, faith-based organizations and leaders, community-based organizations, community pharmacies, schools, traditional birth attendants, and health promotion officers.

### Recommendation 3: Integrate hypertension control/management programs with existing HIV education within the current community and community health extension workers’ training programs

This can be done by including the Nigerian Simplified Hypertension Protocol into the professional training of community and community health extension workers in their different school curriculums, as a capacity building hypertension management program. This will ensure that non - physician healthcare providers who are key in this integration are well trained prior to working in these clinics.

### Recommendation 4: Expand accessibility of national and state health insurance schemes

This can be done by including educational components into the national insurance program. This can be done by organizing community education sessions (e.g. at religious settings or town meetings) to encourage community members to sign up for free or cost-effective health care through the scheme. This scheme can be promoted through the current HIV advocacy and support group programs as well.

### Recommendation 5: Integrate Hypertension Management into Public Primary Health Care in Akwa Ibom

Local policymakers should leverage the early gains of our on-going study regarding the training of community nurses working in the primary care facilities on the management of hypertension to integrate hypertension care into public primary healthcare in Akwa Ibom State.

## Data Availability

The datasets used and/or analyzed during the current study are available from the corresponding author upon reasonable request.

## LIST OF ABBREVIATIONS

AIDS: Acquired Immune Deficiency Syndrome
HIV: Human Immunodeficiency Virus
MAP-IT: Managing Hypertension Among People Living with HIV: an Integrated Model
NCD: Non-Communicable Diseases
PHC: Primary Health Center
PLHIV: People Living with HIV

## References

[1] Onovo A.A., , Estimation of HIV prevalence and burden in Nigeria: a bayesian predictive modelling study. EClinicalMedicine, 2023. 62. 10.1016/j.eclinm.2023.102098 PMC10393599 37538543

[2] Negin J., , Aging with HIV in Africa: the challenges of living longer. Aids, 2012. 26: p. S1–S5. 22713477 10.1097/QAD.0b013e3283560f54 PMC4017661

[3] Aifah A.A., , Study design and protocol of a stepped wedge cluster randomized trial using a practical implementation strategy as a model for hypertension-HIVintegration—the MAP-IT trial. Implementation Science, 2023. 18(1): p. 1–12. 37165382 10.1186/s13012-023-01272-5 PMC10173657

[4] Aifah A.A., , Integration of a task strengthening strategy for hypertension management into HIV care in Nigeria: a cluster randomized controlled trial study protocol. Implementation Science, 2021. 16(1): p. 1–13. 34789277 10.1186/s13012-021-01167-3 PMC8597211

[5] Ojji D., , Building Capacity of Community Nurses to Strengthen the Management of Uncomplicated Hypertension in Persons Living with HIV in Low-and Middle-Income Countries. Global Heart, 2023. 18(1). 10.5334/gh.1216 PMC10348068 37457321

[6] Udonquak A., Akwa Ibom launches sensitisation campaign on health insurance scheme enrollment, in BusinessDay. 2023.

[7] Davis K., , Association between HIV infection and hypertension: a global systematic review and meta-analysis of cross-sectional studies. BMC medicine, 2021. 19: p. 1–16. 33980222 10.1186/s12916-021-01978-7 PMC8117497

